# Blocking and being blocked on gay dating apps among MSM attending a sexual health clinic: an observational study

**DOI:** 10.1186/s12889-021-12182-w

**Published:** 2021-11-19

**Authors:** Navin Kumar, Laura Forastiere, Kamila Janmohamed, Tiange P. Zhang, Yongjie Sha, Fei Yu, Ligang Yang, Joseph D. Tucker, Weiming Tang, Marcus Alexander

**Affiliations:** 1 Yale School of Medicine, Yale University, New Haven, CT USA; 2 Department of Biostatistics, Yale School of Public Health, New Haven, CT USA; 3 University of North Carolina at Chapel Hill Project-China, No. 2 Lujing Road, Guangzhou, 510095 China; 4 Loyola University Chicago Stritch School of Medicine, Maywood, IL USA; 5 Blued.com, Beijing, China; 6 Southern Medical University Dermatology Hospital, Guangzhou, China; 7 School of Medicine, University of North Carolina at Chapel Hill, Chapel Hill, NC USA; 8 Faculty of Infectious and Tropical Diseases, London School of Hygiene and Tropical Medicine, London, UK

**Keywords:** Men who have sex with men, Dating apps, Blocking, HIV, Social networks

## Abstract

**Background:**

There are limited studies on blocking and men who have sex with men (MSM) health outcomes. We need such data in China, to better understand the relationship between Chinese MSM gay app use and health outcomes, thus providing insight on risky sexual behaviors and HIV transmission among Chinese MSM - one of the world’s largest MSM communities. Blocking someone is when users select a function on an app to prevent another user from contacting them and being blocked is when someone is prevented from contacting another user. We studied the correlates of blocking on the world’s largest gay dating app among Chinese MSM (*N* = 208).

**Methods:**

We conducted a cross-sectional survey as part of an HIV testing intervention in Guangzhou, China, May–December 2019. Using logistic regression models, we estimated the correlates of blocking (e.g. sociodemographic characteristics, sexual behavior, HIV testing history, social network data).

**Results:**

MSM had a mean age of 27.9 years (SD = 7.1) and median of one sexual partner in the last 3 months. About 62% had blocked someone in their lifetime and 46% had been blocked in their lifetime. Each additional male partner was associated with an 87% (aOR = 1.87, 95%CI = 1.03, 3.40) increased chance of being blocked. Reporting a versatile sexual role was related with a 90% (aOR = 0.10, 95%CI = 0.02, 0.45) decreased likelihood of blocking behavior and an 86% (aOR = 0.14, 95%CI = 0.04, 0.46) reduced chance of being blocked.

**Conclusions:**

Number of male partners may be associated with blocking behavior, with implications for the design of online sexual health interventions.

**Supplementary Information:**

The online version contains supplementary material available at 10.1186/s12889-021-12182-w.

## Introduction

Although most men who have sex with men (MSM) in China do not disclose their sexual orientation to anyone [1], there is a rich and expanding digital social life for these men [2, 3]. The world’s largest gay social networking app, Blued, is based in China and provides news, health services, shopping, and gaming [2, 3]. MSM spend an average of 80 min a day on such apps [4]. Blocking, both being blocked and blocking someone, are social network ties produced when MSM use gay apps. Blocking someone is when users select a function on an app to prevent another user from contacting them and being blocked is when someone is prevented from contacting another user [5]. A social network tie is defined as any kind of relation between two individuals [6]. When someone blocks another or gets blocked, we can interpret this as a negative or antagonistic social network tie [7]. Users may block others because of harassment, lack of attractiveness, screening for time-wasters, racism (individuals blocking users who might be/have been racist towards them), perceived HIV risk, privacy and safety concerns [5, 8–10]. Blocking can be a protective behavior for the person doing the blocking and also a negative behavior associated with increased anxiety, distress, isolation for the person who was blocked [5, 8].

While blocking may have protective aspects, it may increase HIV risk among MSM by facilitating the formation of segregated groups with greater sexual risk when men block others as a protective mechanism against racism and discrimination [9]. Overall, blocking behaviors can cascade through social networks [11, 12] and thus the correlates of blocking warrant future study. App-based health interventions among MSM are becoming increasingly common [13, 14], but several features on such apps, such as blocking, and their public health implications are not well understood. Such understudied features may have unintended consequences on MSM sexual networks and HIV risk [9]. There are about 780,000 people living with HIV in China [15], with sexual contact being the most common mode of transmission [16]. Chinese MSM are a high-risk population for HIV infection [17] and there are about 21 million MSM in China [18]. Moreover, the HIV prevalence is swiftly rising, from 0.9% in 2003 to 7.3% in 2013 [17].

There are limited studies on blocking and men who have sex with men (MSM) health outcomes [8, 9]. Health outcomes are defined as any measure that directly captures the state of a person’s health [19]. We need such data in China, to better understand the relationship between Chinese MSM gay app use and health outcomes [20]. Such studies of gay apps may provide insight on risky sexual behaviors and HIV transmission among Chinese MSM - one of the world’s largest MSM communities [20, 21]. Risky sexual behavior is defined as behavior that increases one’s risk of contracting or being infected by sexually transmitted infections (STIs) [22]. More insight on how we may improve health outcomes among Chinese MSM is key to addressing the global HIV burden [23]. We conducted an exploratory analysis to explore the factors (e.g. sociodemographic characteristics, sexual behavior, HIV testing history, social network data) that may contribute to blocking behavior, allowing us to better understand blocking and how we may thus alleviate the global HIV burden. Factors were chosen based on their relationship with risky sexual behaviors among MSM [24–26].

## Methods

### Study design and participants

We conducted a randomized controlled trial among MSM in Guangzhou, China that sought to promote male partner testing through social network-based distribution of HIV self-test (HIVST) kits in a cohort study. Participants were offered HIV self-test kits and asked to distribute kits to their social network contacts [27]. Our sample size was estimated with a power of 0.90, an alpha of 0.05, and a lost to follow-up rate of 0.20. More information on sample size calculations is provided in the study protocol [27]. In the randomized controlled trial, enrolled MSM were randomly assigned to one of three groups: 1) standard secondary distribution, 2) secondary distribution with monetary incentives arm, and 3) secondary distribution with monetary incentives plus peer referral [27]. We used data from 1) and 2).

MSM were recruited from May 2019 to December 2019 through a social media account run for MSM-centric studies via posts within the account, and through an MSM-friendly clinic at the Guangdong Provincial Center for Skin Diseases and Sexually Transmitted Infection (STI) Control via approaching participants who came for STI testing. MSM were first recruited for the treatment arms and once recruitment for the treatment arms had been completed, MSM were recruited for the control arm. Participants interested in HIV testing at the clinic could book appointments online or enroll in the study at the clinic. MSM were screened for the following criteria: 1) aged *≥*18 years; 2) presumed male at birth (transgender women were not able to participate in the study); 3) ever had sex with men; 4) willing to be surveyed at baseline and followup. Eligible participants were provided with study information, such as, potential risks, benefits, procedures, and outcomes. Informed consent was obtained from all subjects.

A baseline survey was administered to eligible participants via a QR code they could scan and thereby complete the survey on their mobile device. The datasets generated and analysed during the current study are not publicly available due to the sensitive nature of the data in the Chinese context but are available from the corresponding author on reasonable request.

#### Survey items

We collected participants’ baseline data such as, sociodemographic characteristics, sexual behavior, HIV testing history, social network data and blocking behavior (survey instrument in Supplement).

##### Independent variables

Sexual behavior items included number of male partners in the last 3 months, and main sexual role. Examples of sexual behavior items were: in the past 3 months, with approximately how many different male sexual partners did you have anal sex? your main sexual role is (pick one) insertive/receptive/both. To represent sexual behavior disclosure to family and medical professional, we used the following item: if you have told others about your sexuality or sexual history with men, who are they? This item had the following options: medical professionals; family members; friends with no sexual relationship; coworkers; employers; other. We recoded the medical professionals option into a binary variable representing sexual behavior disclosure to medical professionals. We similarly recoded the family members option to a binary variable representing sexual behavior disclosure to one’s family. To represent condom use, the following was used: In the last 3 months, how often did you use condoms during anal sex with men? with the options: never used; occasionally (less than half of the time); often used (more than half of the time); every time. Income was measured with the following item: How much is your monthly income? The following options were provided for this question: less than USD5,171; USD5,171 - USD10,342; USD5,171 - USD10,342; More than USD17,236.

Social network survey items included name generator and descriptor questions to measure social network degree (number of people whom you have a social tie to) and weighted social network degree (social network degree weighted by the frequency of contact) [28]. Social network degree was based on the sum of people listed in the question: Besides your family members, who are the people you spend your free time with? (list up to five). For example, if someone listed four people to the indicated question, their assigned social network degree was four. Weighted social network degree was based on the following item: How often do you contact the indicated person? This item had the options: once a year; once every 6 months; once a month; once a week; daily. We treated this as an ordinal variable (scale of 1–5) and summed the item across each social contact listed in the social network degree question. For example, if someone had a social network degree of four and contacted each person once a month, the weighted social network degree was 3 + 3 + 3 + 3 = 12. These items were based on validated instruments used to measure social network characteristics [28]. We note that our method does not distinguish between five contacts with a contact frequency of once a year and a single contact one sees daily. However, our goal was to measure the magnitude of one’s social connectedness, not calculate the total number of social connections one had.

##### Dependent variables

Blocking items included likelihood of engaging in blocking, both directed and undirected behavior, and level of distress caused by being blocked. Directed blocking behavior refers to blocking behavior that was targeted i.e. person A blocked person B (being blocked) or person A got blocked by person B (being blocked). Undirected blocking behavior is the sum of total blocking behaviors i.e. if person A blocked someone and also reported being blocked, they would have a total of two undirected blocking behaviors in that time period. Examples of questions were: Have you ever blocked someone on a Blued? Have you ever been blocked by someone else on Blued?

### Ethical review

Participant anonymity was maintained during the entire project. No identifying information was collected. IRB approval was obtained from the Dermatology Hospital of Southern Medical University (GDDHLS-20180503) and the University of North Carolina at Chapel Hill (18–1358). All methods were carried out in accordance with relevant guidelines and regulations.

### Statistical analysis

We calculated descriptives (Table 1) using data obtained from all MSM surveyed.

**Table 1:** Participant characteristics for 208 Chinese MSM, collected in a randomized controlled trial in Guangzhou, China

Variable	Mean (SD)
Age	27.9 (7.1)
	missing = 0%
Number of male partners in the past 3 months	median = 1
	missing = 42%
Social network degree	2.3 (1.1)
	missing = 0%
Weighted social network degree	8.5 (4.2)
	missing = 0%
	%
*Yearly income (USD/year)*	
Less than USD5,171	20.7
USD5,171 - USD10,342	35.1
USD10,342 - USD17,236	30.8
More than USD17,236	13.5
	*n* = 208
	missing = 0%
*Condom use*	
Never used	5.7
Occasionally (Less than half of the time)	7.4
Often used (More than half of the time)	24.6
Every time	62.3
	*n* = 122
	missing = 41%
*Sexual behavior disclosure to family*	
Yes	22.9
No	77.1
	*n* = 166
	missing = 20%
*Sexual behavior disclosure to medical professional*	
Yes	66.3
No	33.7
	*n* = 166
	missing = 2-%
*Prior HIV test*	
Yes	83.7
No	16.4
	*n* = 208
	missing = 0%
*Sexual role*	
Insertive	42.6
Receptive	20.5
Versatile	36.9
	*n* = 122
	missing = 41%
*Undirected blocking behavior*	
Yes	74.6
No	25.4
	*n* = 181
	missing = 13%
*Blocked by someone*	
Yes	46.4
No	53.6
	*n* = 181
	missing = 13%
*Blocked someone*	
Yes	62.4
No	37.6
	181
	missing = 13%

Conversion rate of RMB 1 = USD 0.14 was used

Missing data on the variables of interest ranged from 0 to 42% (see Table 1). To account for nontrivial missing data, multiple imputation was conducted using multiple imputation with chained equations (MICE) to fill missing values with logistic regression imputation methods [29]. Data was assumed to be missing at random (MAR) and we imputed values based on key independent and dependent variables included in the final model. Our decision to categorize data as MAR was based on the fact that the data missing was systematically related to the observed but not the unobserved data. We did not find any evidence that data was missing not at random (MNAR) [30]. For example, we did not find that individuals with lower income were more likely to have missing income data.

Forty-two imputed datasets were generated to ensure the number of imputed datasets was at least as large as the percentage of incomplete information for the main outcomes or the parameter-specific fraction of missing data for all parameters used in the final models, whichever was higher [31]. We performed numerous model diagnostics. With subject matter expertise, we examined graphical displays of the imputed data using histograms and conducted similar analysis by generating descriptive statistics [32].

We then compared observed and imputed data using density plots and cumulative distribution plots [32, 33]. We also compared means and variances of observed and imputed values [34]. Finally, we generated goodness-of-fit of the imputation models using established methods for checking assumptions of regression models [33].

We modeled blocking behavior (undirected blocking behavior, blocking someone, being blocked) using multivariate logistic regression and reported adjusted odds ratio estimates for each independent variable. We used a multivariate regression instead of bivariate analyses as blocking behavior is complex and involves socio-demographic characteristics and sexual behavior [5, 8–10]. A multivariate regression is more appropriate when numerous independent variables are associated with an outcome [35]. We conform to best practice in previous work in this area, which utilized multivariate regression [36, 37]. Similarly, to better understand blocking and its associated covariates, we need to predict blocking instead of investigating it as an exposure which indicates sexual risk or mental health issues.

We adjusted for the following factors and intervention assignment. We decided on these factors based on guidelines established in other studies with the same population [36, 37]. We understand the issues around more complex models vs simpler models and have selected the current variables in accordance with best practice as per our previous work in this topic [36, 37]. Independent variables represented sexual behavior [sexual role, number of male partners in the past three months, condom use, disclosure of MSM sexual behavior to family, disclosure of MSM sexual behavior to medical professional, prior HIV test], participant social network structure (social network degree, weighted social network degree), and sociodemographic categories (income, age). The Income variable was denominated in the survey as RMB/month and we converted it to USD/year for clarity. Social network degree and weighted social network degree were calculated as indicated in the Survey Items section, and all other variables were used unaltered from the survey instrument. Analysis was conducted in R [38].

## Results

We were unable to provide STI prevalence as this will be the purview of the main randomized controlled trial [27].

### Sociodemographic characteristics

Two hundred and eight MSM enrolled in the study. We presented descriptive statistics in Table 1. MSM had a mean age of 27.9 years (SD = 7.1) and median of one sexual partner in the last 3 months. Participants had a mean of 2.3 social ties (SD = 1.1) and a mean weighted social network degree of 8.5 (SD = 4.2). MSM generally fell into two yearly income groups; USD5,171 - USD10,342 (35.1%) and USD10,342 - USD17,236 (30.8%). In the last 3 months, most MSM (62.3%) used condoms every time during anal sex with men. Most had not disclosed sexual behavior to their family (77.1%), but had disclosed sexual behavior to their medical professional (66.3%). Most had a prior HIV test (83.7%). Blocking was a common behavior. Most MSM (75%) had engaged in undirected blocking behavior in their lifetime i.e. they had blocked someone or had been blocked. About 62% had blocked someone in their lifetime and 46% had been blocked in their lifetime.

### Multivariate analyses of blocking correlates among Chinese MSM

We presented multivariate analyses in Table 2. Only undirected blocking behavior and blocked by someone (being blocked) were presented as only these yielded statistically significant results for associations with independent variables. The effect of all results are to be interpreted as being controlled for earlier indicated independent variables. A greater income was associated with a 2% (aOR = 1.02, 95%CI = 0.50, 2.05) increased chance of undirected blocking behavior. Reporting a versatile sexual role was related with a 90% (aOR = 0.10, 95%CI = 0.02, 0.45) decreased likelihood of blocking behavior and an 86% (aOR = 0.14, 95%CI = 0.04, 0.46) reduced chance of being blocked. Each additional male partner was associated with an 87% (aOR = 1.87, 95%CI = 1.03, 3.40) increased chance of being blocked. Results without MICE are indicated in Supplementary Table 1.

**Table 2:** Multivariate results of the relationship between blocking behaviors and income, age, sexual role, number of male partners in the past 3 months, condom use, sexual behavior disclosure, and prior HIV test

Variable	aOR	P	(95% CI)	aOR	P	(95% CI)
Undirected blocking behavior Blocked by someone						
Income	1.02	*p <* 0.01	(0.50, 2.05)	0.71	0.30	(0.37, 1.37)
Age	0.98	0.70	(0.88, 1.09)	1.02	0.72	(0.93, 1.12)
Sexual role						
Insertive	–	–	–	–	–	
Receptive	0.20	0.06	(0.04, 1.06)	0.40	0.15	(0.12, 1.42)
Versatile	0.10	*p <* 0.01	(0.02, 0.45)	0.14	*p <* 0.01	(0.04, 0.46)
Number of male partners in the past 3 months	2.02	0.07	(0.94, 4.34)	1.87	0.04	(1.03, 3.40)
Condom use	0.98	0.93	(0.49, 1.92)	0.82	0.51	(0.45, 1.50)
Social network degree	0.05	0.94	(0.29, 3.83)	1.29	0.65	(0.43, 3.92)
Weighted social network degree	1.05	0.77	(0.76, 1.46)	0.90	0.44	(0.68, 1.19)
Sexual behavior disclosure to family	2.66	0.17	(0.65, 10.82)	0.67	0.38	(0.52, 5.30)
Sexual behavior disclosure to medical professional	0.97	0.93	(0.49, 1.92)	0.06	0.92	(0.36, 3.10)
Prior HIV test	1.98	0.48	(0.29, 13.49)	7.90	0.05	(0.99, 63.13)
Intervention	1.83	0.31	(0.57, 5.86)	1.15	0.80	(0.40, 3.40)
N		208			208	

We estimated all aORs with logistic regression models. Adjusted Odds Ratios account for the above independent variables and intervention assignment, *aOR* Adjusted odds ratio

## Discussion

Our exploratory analysis detailed that having more male partners was associated with an increased likelihood of getting blocked. However, given our limited data, it is also plausible that those with higher activity in the app may have more partners and get blocked more often. There is limited empirical research on blocking [8, 9], and none on the public health implications of blocking. It is possible that unrestricted sociosexuality, and short-term mating orientation, is related to higher dating app use and, thus a higher probability of blocking/being blocked. Studies on blocking are predominantly conducted in high income nations, but not in low- and middle-income nations such as China, where there is a large MSM HIV burden [39]. Men with more male partners may be viewed as promiscuous or having STIs [40]. Upon finding out this information, other men may decide to block individuals with more partners. We also note that other explanations are available e.g. those with more partners use the apps more often and due to higher activity their probability of being blocked is also higher.

Overall, with more detailed epidemiological data, we suggest that future work expand on the correlates of blocking. While we are uncertain on the direction of causality, designing interventions on gay dating apps to target certain behaviors and demographic groups may ensure that blocking is mostly used for positive purposes such as limiting racism and discrimination [8, 9] without creating self-segregated groups with greater HIV risk. An example of such an intervention may be cautioning users who block excessively within a certain time frame, a technique previously used on social media to reduce racism [41]. Users can be sent a message indicating that while blocking can have protective effects, excessive use may not be advisable. Mitigating excessive blocking may reduce the likelihood of MSM forming closed groups with greater HIV risk [8, 9], keeping in mind the benefits of sexual exclusivity within a certain group [42].

### Limitations

Several limitations were due to the design of the randomized controlled trial. Due to the online recruitment strategy, we may have overlooked individuals who cannot access the internet. In the online surveys, behaviors of participants were self-reported, which may increase the possibility of social desirability bias [27]. We used sequential enrolment due to limited resources, and note possible effects of this enrolment strategy on the results. Future work will assign MSM to both arms randomly and at the same time. We were also unable to record the proportion of eligible MSM who actually accepted to be part of the study. Unmeasured factors, such as time of blocking event and reciprocal blocking may have driven our results. We were unable to collect data on the number of blocks made/received due to restrictions in the survey length and limited resources. We were also unable to collect time spent using the app due to privacy restrictions. The survey did not provide a description of what was meant by sex with a woman and there was no capture of people who may have been assigned female at birth but now identify as male.

Participants may have reported lower amounts of blocking than experienced, and over-reported blocking someone. We were unable to control for such effects but plan future study to use data drawn directly from apps rather than relying on participant self-report. We did not consider the possibility of imputing separate datasets based on a stratification variable e.g. enrolment location, recruiter type, and will include such techniques in future research. It is possible our results were driven by MSM making more attempts at contacting others, thus increasing the rejections (getting blocked) they are likely to receive. Thus, future qualitative work can detail the underlying factors behind our results. Studies of online rejection - including but not exclusively in the context of racism - have found that the effects of sexual health are mediated by the effects of those experiences on mental health [43, 44]. Future work can thus explore how mental health is related to blocking and sexual health outcomes. Data was collected at sites catered to MSM STI testing. Such site selection may have limited our sample to MSM connected with community-based organizations and perhaps more likely to engage in sexual behavior disclosure. Generalizability of findings may be limited outside the Chinese MSM context.

## Conclusions

Number of male partners may be associated with blocking behavior, with implications for the design of online sexual health interventions.

## Supplementary Information

**Additional file 1: Supplementary Table 1.** Multivariate results of the relationship between blocking behaviors and income, age, sexual role, number of male partners in the past three months, condom use, sexual behavior disclosure, and prior HIV test without imputation.

## Data Availability

The datasets used and analyzed during the current study available from the corresponding author on reasonable request.
