# Supplementary material for: Blocking and being blocked on gay dating apps among MSM attending a sexual health clinic: an observational study
Source: BMC Public Health. 2021 Nov 19;21:2127. doi: 10.1186/s12889-021-12182-w (PMC8605500; doi:10.1186/s12889-021-12182-w)
Supplement: Supplementary file 1 — Additional file 1: Supplementary Table 1. Multivariate results of the relationship between blocking behaviors and income, age, sexual role, number of male partners in the past three months, condom use, sexual behavior disclosure, and prior HIV test without imputation. [file 12889_2021_12182_MOESM1_ESM.docx]

20

**Supplementary table 1:** Multivariate results of the relationship between blocking behaviors and income, age, sexual role, number of male partners in the past three months, condom use, sexual behavior disclosure, and prior HIV test without imputation

| Variable | aOR | P | (95% CI) | aOR | P | (95% CI) |
| --- | --- | --- | --- | --- | --- | --- |
| Undirected blocking behavior Blocked by someone | | | | | | |
| Income | 1.02 | p*<*0.01 | (0.32, 1.71) | 0.71 | 0.03 | (0.06, 1.36) |
| Age | 0.98 | p*<*0.01 | (0.87, 1.09)) | 1.02 | p*<*0.01 | (0.92, 1.11) |
| Sexual role |  |  |  |  |  |  |
| Insertive | - | - | - | - | - |  |
| Receptive | 0.20 | 0.82 | (-1.45, 1.62) | 0.40 | 0.53 | (-0.86, 1.65) |
| Versatile | 0.10 | 0.90 | (-1.42, 1.62) | 0.14 | 0.81 | (-1.00, 1.29) |
| Number of male partners in the past three months | 2.02 | p*<*0.01 | (1.26, 2.77) | 1.87 | p*<*0.01 | (1.29, 2.46) |
| Condom use | 0.97 | p*<*0.01 | (0.30, 1.64) | 0.82 | 0.01 | (0.23, 1.41) |
| Social network degree | 1.05 | 0.11 | (-0.22, 2.32) | 1.29 | 0.02 | (0.20, 2.38) |
| Weighted social network degree | 1.05 | p*<*0.01 | (0.73, 1.37) | 0.90 | p*<*0.01 | (0.62, 1.17) |
| Sexual behavior disclosure to family | 2.66 | p*<*0.01 | (1.28, 4.04) | 1.67 | 0.01 | (0.53, 2.80) |
| Sexual behavior disclosure to medical professional | 1.35 | 0.02 | (0.21, 2.50) | 1.06 | 0.05 | (0.00, 2.12) |
| Prior HIV test | 1.98 | 0.04 | (0.09, 3.87) | 7.90 | 0.00 | (5.86, 9.95) |
| Intervention | 1.83 | p*<*0.01 | (0.68, 2.98) | 0.41 | 0.83 | (0.11, 2.18) |
| N |  | 94 |  |  | 94 |  |

Note: We estimated all aORs with logistic regression models. Adjusted Odds Ratios ac- count for the above independent variables and intervention assignment. aOR=adjusted odds ratio.
